# Implementation of an App Based Communication Platform, “Consultant Connect", to Improve Physical Health Outcomes for Patients at a UK Mental Health Trust

**DOI:** 10.1192/bjo.2022.402

**Published:** 2022-06-20

**Authors:** Isabel McMullen, Raymond McGrath, Karen Ang, Elli Fairbairn, Julie Williams, Prashanth Reddy, Nick Sevdalis, Fiona Gaughran

**Affiliations:** 1 South London and Maudsley NHS Foundation Trust, London, United Kingdom; 2 King's College London, London, United Kingdom; 3 King's College Hospital, London, United Kingdom

## Abstract

**Aims:**

Patients with mental health disorders are known to have worse physical health outcomes. ‘Consultant Connect’ (CC) is an app-based communication platform which aims to improve patient outcomes and experience, by offering clinicians direct access to consultants working in a partnership acute Trust, so they can seek advice and guidance for their patients’ physical health problems. This creates whole system efficiencies by avoiding unnecessary referrals to an Emergency Department or outpatient clinics. This poster describes the implementation of CC in a large UK Mental Health Trust. Initially designed for GPs, this is the first time a UK Mental Health Trust has used CC.

**Methods:**

Consultant Connect was launched in the Mental Health Trust's inpatient services in June 2020 as part of a Trust-wide programme of work aiming to improve the physical healthcare of mental health patients. In July 2021 it was rolled out across all services, including all community services. All platform activity was monitored and the implementation team collected data to determine: a) origin of call, b) which specialty was required, c) numbers of calls successfully connected, and in a subset of calls d) outcome of call. In addition, 183 call recordings were analysed, to identify clinical training needs and inform further development of the platform.

**Results:**

In the period June 2020 – December 2021, there were 1422 use episodes of the CC platform by Mental Health Trust clinicians. There were 401 Trust registered downloads of the CC App by the Trust clinicians. 53 different clinical specialties were contacted, with cardiology (414 calls), diabetes and endocrinology (243 calls), and haematology (124 calls) the most frequently called. 68% of queries received a response. 48% of calls had an outcome recorded, with 70% of these resulting in the physical healthcare being delivered by the mental health team, following the advice received (i.e. referral or admission avoided, or the patient treated out of hospital).

**Conclusion:**

CC is being progressively embedded into clinical practice and has become a well-used pathway for mental health clinicians seeking immediate clinical advice from acute hospital Consultant colleagues. Further qualitative and quantitative work is planned with mental health clinicians, patients and carers to better understand their experience and determine if it improves care from both the clinicians’ and patients’ perspective.

